# A Literature Review on Equine Bedding: Impacts on Horse and Human Welfare, Health, and the Environment

**DOI:** 10.3390/ani15050751

**Published:** 2025-03-05

**Authors:** Naod Thomas Masebo, Beatrice Benedetti, Maria Mountricha, Leonie Lee, Barbara Padalino

**Affiliations:** 1 Department of Agricultural and Food Sciences, University of Bologna, Viale Fanin 46, 40127 Bologna, Italy; naodthomas.masebo2@unibo.it (N.T.M.); beatrice.benedetti7@unibo.it (B.B.); maria.mountricha@unibo.it (M.M.); 2 Equined, Geelong, VIC 3220, Australia; leonie@equined.com.au; 3 Faculty of Science and Engineering, Southern Cross University, Military Road, Limore, NSW 2480, Australia

**Keywords:** One Welfare, well-being, sustainability, straw, wood shavings

## Abstract

Bedding is an important component of equine accommodation management. Choosing the right bedding is important for stable management and its selection may include considerations such as the sourcing of the material, the capital investment and ongoing costs, delivery, storage, installation, ongoing labour and maintenance, removal and disposal. Furthermore, it is crucial that the consequences for the health and welfare of horses and humans and the impact on the environment should also be considered. This review aimed to outline the advantages and disadvantages of different horse bedding types, focusing on their effects on the well-being of horses, humans, and the environment. The Preferred Reporting Items for Systematic Reviews and Meta-Analyses (PRISMA) technique was used as the methodology for this review. The search was performed in Scopus and Web of Science bibliometric databases and a total of 176 records were screened reading the title and the abstract. After screening, 58 records were retained and another 19 records were identified using their reference lists (i.e., snowballing). Therefore, a total of 77 records were considered. Straw and wood shavings were the most commonly used and studied bedding materials, while research on alternative options remains limited. Straw is identified as horses’ preferred option, while shavings appear to be the easiest to clean, making them the preferred choice for stable workers. The parameters to consider when choosing the bedding most fit for purpose are many and their attributes differ across the various bedding types. This review has compared all the bedding types within the research literature to determine the best overall option using the research-based evidence. Each bedding type offers unique benefits and drawbacks summarised in a user-friendly table. Stable managers must consider and evaluate them to suit their specific needs, including the health and welfare of each horse and the husbandry system involved. Our findings may, therefore, be useful in the decision-making process of equine industry members.

## Simple Summary

Bedding describes the range of materials usually placed on the floor of the horse’s accommodation, mainly to absorb urine and moisture from manure and provide a comfortable and dry environment. However, little is known about how most bedding materials affect the welfare of horses and stable workers as well as its environmental impact. Therefore, the aim of this review was to describe the main characteristics of different bedding materials, focusing on their effects on the well-being of horses, humans, and the environment. Of all the different types, straw and wood shavings are the most commonly used. For this reason, they are also the most studied in terms of their impact on animal and human health and welfare. Straw seemed to be the preferred choice for horses, as it is comfortable, edible and promotes recumbency. However, as straw is edible and may be a source for dust, it has the potential to contribute to health problems in both horses and stable workers. Wood shavings vary substantially in quality, and only the de-dusted and dehydrated varieties offer benefits. Other types of bedding such as pellets, paper-based bedding and sand are less commonly used and fewer studies have been conducted on them. This review confirms that identifying a single optimal bedding material or system is challenging, as each option presents distinct advantages and disadvantages that must be evaluated in the context of the specific situation. Our findings, summarising the pros and cons of each bedding, are therefore useful for the equine industry to inform their decision-making process when it comes to the selection of bedding for their horse accommodation.

## 1. Introduction

Bedding material is important for the daily management of stabled horses. It provides different functions in horse stables such as absorbing the moisture from the horses’ excretions, creating the ideal microclimate conditions for resting and an insulation layer that prevents the loss of heat through the floor [1]. The type of bedding material used significantly contributes to the hygiene and comfort of horse stables [2]. Hence, the choice of the right bedding material promotes the health and welfare of horses [2].

The absorbing nature of the bedding material is an important factor to consider, and it should be highly absorbent to optimise a dry environment and prevent the development and accumulation of harmful substances in the stable that can impair the health of horses and humans [3]. Furthermore, the bedding material should provide comfort for the horses to rest, promoting recumbent sleep and so enhancing their welfare [4]. In addition, the bedding material facilitates and encourages horses to urinate in their accommodation since the urine can be absorbed by the bedding material on impact and reduces the potential for splash on the horses’ legs [5]. From a safety point of view, the bedding should be adequately deep, with a depth depending on the type of bedding material [6]. The quality of bedding material also affects the air quality inside the stable and airborne contaminants can have a negative impact on the respiratory tract of horses and personnel working in the stables [1]. Finally, the cushion properties of the bedding material are important [3,7] and bedding should be soft enough to protect the horse from injuries and pressure sores potentially caused by contact with the floor base material beneath.

To keep a horse healthy and comfortable in the stables, bedding material, such as straw and wood shavings, should be managed properly including removing faeces, wet bedding and soiled feed as required [8]. Bedding material is frequently replaced to ensure the horse has a hygienic, clean and dry environment to rest [5,8]. The bedding material can be managed differently depending on its type. Management includes ventilation/aeration of the whole bedding material and frequent removal of faeces and urine [9]. For instance, some studies have focused on the effects of mucking out and suggested that when performing mucking out horses should not be kept inside the stable due to the increase in airborne contamination, and full mucking should not be performed on a daily basis [8].

A variety of materials can be used as bedding, such as wood shavings, straw, sawdust, compressed wood pellets and rubber matting [10]. Alternative options for bedding materials have been introduced to the market due to factors such as the increasing price of straw and wood shavings coupled with a reduction in their quality. These include peat moss, cardboard, rice hulls, sand, coconut, flax, hemp, and shredded paper [9]. Different materials such as biocompost have been tried out as bedding but were not recommended due to contributing to poor air hygiene and resulting in equine respiratory health problems including obstruction of the airways. Other materials such as wood fiber sludge, have been proposed as horse bedding mainly due to an enforced law which enforces circular economy and recycling of the by-product of the pulp and paper industry [11]. However, in the study of Seedorf et al. [12], the authors highlighted the importance of adequately testing any new bedding material to determine any welfare implications for the horses and stable workers prior to introducing the material to the market for use in equine accommodation.

Generally, the bedding material used in stables is chosen or recommended based on a number of criteria, including availability, cost, ease of use, and the ability to clean the horse’s accommodation quickly and efficiently. It should also optimise a healthy stable environment for the workers and the horses. This includes minimising dust and allergens, particularly in relation to the dustiness of the bedding, and limiting the production of gaseous ammonia, as per the water-binding capacity of the bedding. The bedding choice should also consider the cost-effective management of the waste manure and whether or not the materials are environmentally friendly [13].

To date, no study has provided a complete overview of the effects of different bedding materials using the One Welfare approach [14]. Therefore, the objective of the current review is to describe the different types of bedding material used in horse stables and to discuss their impact on the well-being of both horses and humans (including the stable workers, riders, equine allied health practitioners and visitors) and the environment (including sustainability and any short-term and/or long-term consequences such as pollution and contamination from its manufacture/processing, use, storage and disposal).

## 2. Materials and Methods

### Search Criteria and Strategy

For this review, the Preferred Reporting Items for Systematic Reviews and Meta-Analyses (PRISMA) method was carried out [15]. Elsevier©’s bibliometric database, Scopus (https://www.scopus.com/home.uri) (accessed 6 January 2025) and Web of Science (https://www.webofscience.com/wos/woscc/advanced-search) (accessed 6 January 2025) were used. The search strings were discussed among the authors and the final string of keywords selected to search the documents in Scopus was agreed upon after various tests were performed to understand which string most suited the purpose of the search.

The final search strings used in Scopus included the following keyword combinations: (horses OR donkeys OR mules OR hinnies OR equine* OR equid* OR equus) W/6 (floor* OR bed*). In Web of Science, the following was used: (TS = ((horses OR donkeys OR mules OR hinnies OR equine* OR equid* OR equus) NEAR/3 (floor* OR bed*))).

The search was based on the documents’ title, abstract, and keywords. Pre-filters were set and these included the publication year (2004–2024), the scientific field (veterinary, agricultural and biological sciences, environmental sciences, and engineering), the document typology (article, review, and book chapter), and the English language. These pre-filters were discussed and agreed upon by the authors and the funding parties, to identify the most recent evidence (i.e., last 20 years) published in selected peer-reviewed journals as per the usual method in several Scientific Opinions of the European Food Safety Authority [16] and the literature [17,18]. The search was conducted on 6 January 2025. The set of records generated was downloaded and put into a single commercial spreadsheet (Microsoft Excel^®^, version 16.0, Redmond, WA, USA). Individual records were arranged in rows in the final dataset, while corresponding information was arranged in columns. The title, abstract, year of publication, authors, corresponding author, affiliations, document type, source of publishing (e.g., journal title), and keywords were specifically recorded for every entry. Two investigators (BB and BP) then reviewed and screened the results by reading the title, the keywords, and the abstract for relevance after having agreed on inclusion and exclusion criteria (Table 1). For example, a manuscript was included when it referenced information and/or data relevant to one or more implications of specific or multiple bedding materials on one or multiple implications for horse and human well-being and the environment [17]. Where it was unclear whether a document should be included or not, the senior author (BP) made the final decision. Many records were excluded as the word/s bedding or floor was used to describe the housing conditions of the horses used in the study, but the aim of the study was not focused on the effects of the bedding and floor. Records without an abstract (n = 3) were also not retrieved. After having identified the records, the full articles were searched, downloaded and evaluated. When the full article was not available (n = 1), the records were also excluded.

Additionally, a snowball search was conducted by the authors by looking at the list of references of the selected articles for retrieving any further records not identified in the initial search but found of interest while reading the full text of the included records [16,17,18].

**Figure 1:**
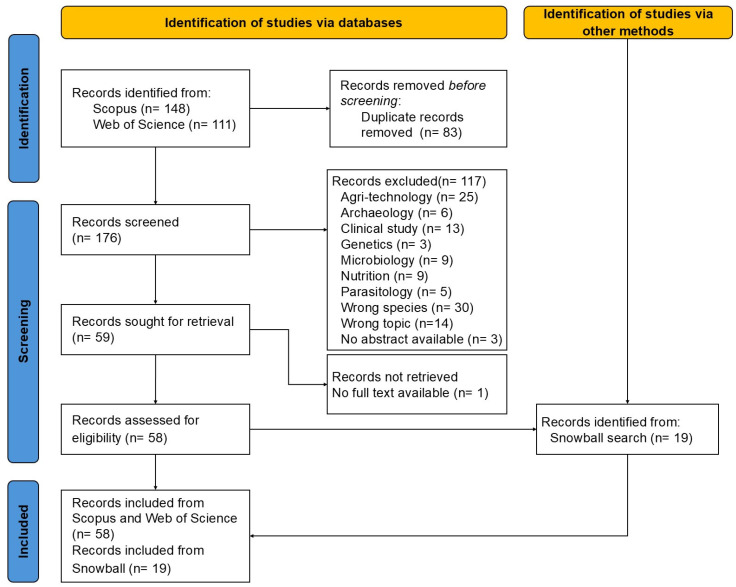
Selection procedure and the total number of records retained (n = 77), the number of excluded records and the exclusion criteria applied in this systematic review of the literature.

In summary, the search revealed a total of 259 records including research articles, reviews, and book chapters and those records were added to the Excel sheet. Out of these, 83 documents were removed due to duplication. Hence, 176 records were screened. A total of 117 articles were excluded and categorised in accordance with the exclusion criteria and, consequently, only 58 records were retained for full-text reading. Additionally, 19 articles were selected by snowballing (Figure 1).

Three authors (NTM, BB and BP) read the full articles and extrapolated the information creating short summaries. Information regarding how the bedding materials impacted human and horse well-being and the environment was extracted. The results are presented in the order of the bedding from the most common to the least.

## 3. Results

### 3.1. Wood-Based Bedding

#### 3.1.1. Wood Shavings and Chips

**Figure 2:**
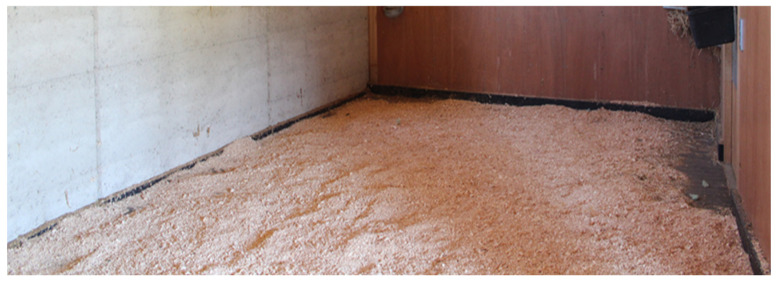
The wood shavings in this stable loose box are of a reduced depth due to the comfort and protection the rubber mattress provides for the horses which cushions the horses from the concrete floor base (Source: Lee’s photo).

**Figure 3:**
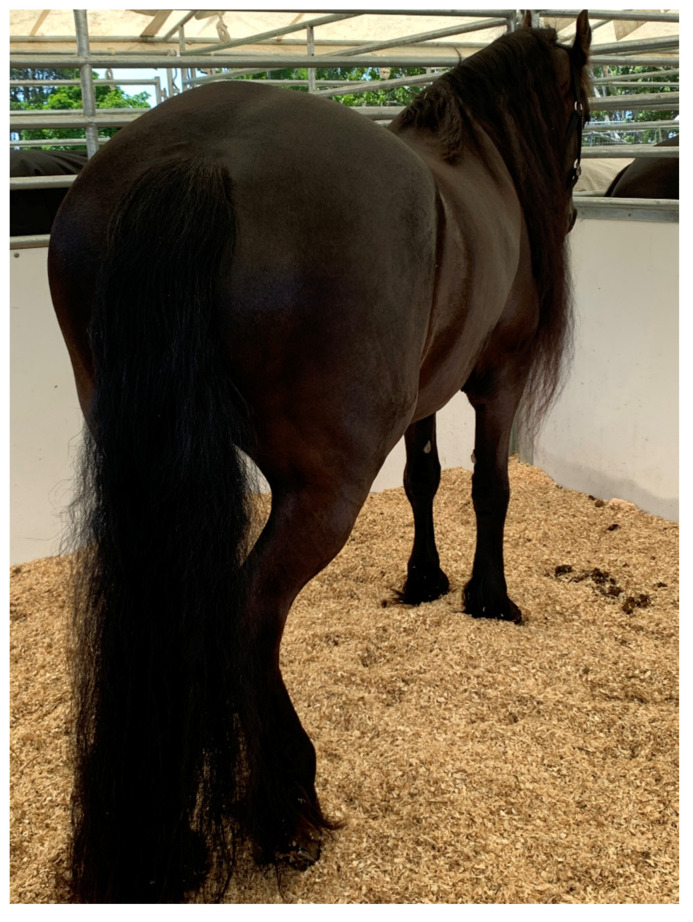
Deep bedding of wood shavings over concrete floor base (Source: Lee’s photo).

**Figure 4:**
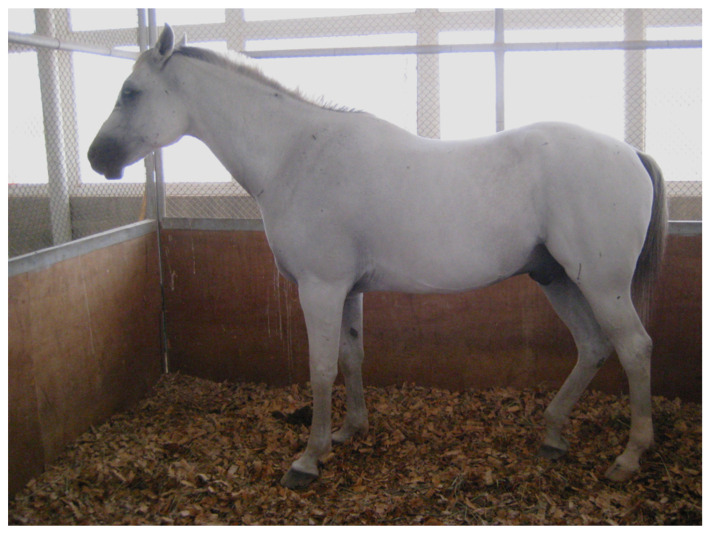
A mix of different-sized shavings form the bedding over the concrete floor base (Source: Lee’s photo).

Wood shavings and chips are generally the waste product obtained from the processing of wood where it is shaped or planed using carpentry tools or machines like planers and milling machines [19] (Figure 2, Figure 3 and Figure 4). Shavings and chips are characterised by 86% dry matter content, and 316% water binding capacity [6]. Together with straw, these are among the most commonly used types of bedding in the horse industry [20]. They are widely used for stabled horses while they are not generally used in paddock resting areas and shelters. Due to their popularity and dissemination, there are a variety of wood shaving options on the market. There is a diversity of plant origins for wood, and it is important to be aware of the plants that are toxic to horses. This includes black walnut shavings, which cause acute laminitis [21].

In terms of quality, wood shavings can be divided into two categories, namely the dust-free/reduced and the not dust-free/reduced shavings. The latter could be very dusty, and it was demonstrated that they can raise the risk of inflammatory airway disease [22]. Accordingly, a study by Monki et al. [23] discovered that horses kept in wood shavings had a larger percentage of neutrophils in their bronchoalveolar lavage fluid (BALF) than horses kept on peat bedding. Conversely, the dust free/reduced type undergoes a process that dried, aired, and cleaned the dust from the shavings. This process lowers the overall dust content and makes it particularly beneficial for horses with respiratory conditions, although all stabled horses would benefit from minimising the dust contamination in the air. These dust-free/reduced wood shavings are produced using standardised processing techniques which make these types of shavings consistent in quality, more absorbent, less dusty and with significantly fewer allergens than other bedding [6]. Another important characteristic of this type of wood shavings is that they provide a dry, safe and clean surface for the horses to stand on, which helps to reduce the risk of bacterial growth in the horses’ hooves and skin irritation [6]. A study conducted by Yarnell et al. [24] showed that Scots pine shavings (Pinus sylvestris) have high antibacterial activity compared with different bedding materials such as Cannabis sativa (hemp) and chopped wheat straw. This study is evidence that some wood shavings have an antimicrobial activity which helps in enhancing the health of the horse. It is also important to consider, especially for horses living in cold climates, that wood shavings are not as warm as other bedding materials such as straw as they do not trap air and provide insulation from the floor base material, and/or the ground and the cold ambient temperature [25]. As cited above, high-quality wood shavings can be expensive, particularly in comparison to straw, but from a management point of view, they are more durable and reduce the mucking out time [26]. In fact, their high water-binding capacity contributes to the need for fewer bedding replacements overall. Dust-free/reduced wood shavings are therefore the preferred bedding option for stables prioritising the respiratory health of horses and stable workers. Wood shavings made of black walnut induce laminitis and should not be used as bedding material in horse stables [27]. With regard to the ammonia concentration, the studies do not all agree, and this possibly confirms that there is an important difference in quality between different types of wood shavings. In particular, some studies report that wood shavings have comparable levels of ammonia to other bedding types: around 1.152 ppm [3]. On the other hand, other studies also reported that the ammonia level in wood shavings is high and can even exceed the recommended level for horses’ health, reaching up to 11 ppm [1,28].

Wood shavings can be considered environmentally friendly, especially when sourced from sustainable, untreated wood and used responsibly. However, it is important to consider factors like the origin of the wood, its processing methods, and the disposal of the bedding after use. Opting for bedding made from recycled or waste wood materials and ensuring proper disposal can make wood shavings a highly eco-friendly choice. In a study, it was shown that the ability of wood shavings to be used as compost can be improved by treating and inoculating them with *A. fumigatus* so they can be efficently used as compost and to improve the compost quality [29]. According to Romano et al. [30], composting horse manure mixed with wood shavings reduces the pathogen content and the authors concluded that the compost can be used to fertilise or mulch around trees or flower gardens or as a soil conditioner on pasturelands, but probably should not be used in growing vegetables.

#### 3.1.2. Sawdust

**Figure 5:**
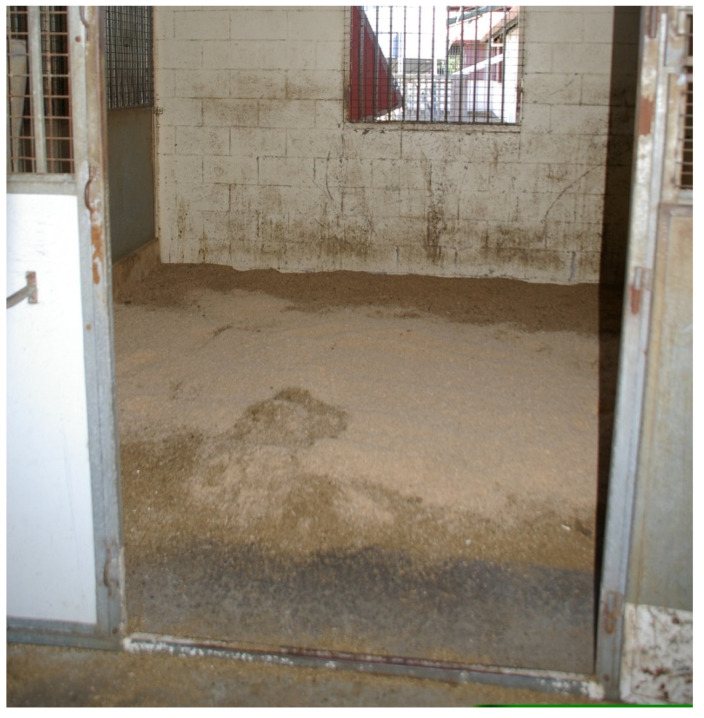
Deep sawdust bedding system on concrete floor base. The darker sections contain greater moisture content which can reduce the airborne contaminants but conversely they may provide a damp bedding environment for horses; this may be problematic for their well-being. (Source: Lee’s photo).

Sawdust is a wood byproduct but, different to wood shavings and chips, it is produced as a result of wood sawing. It is readily available in many places, but it is most prevalently used in Northern European nations [31]. Moreover, it is cheaper than wood shavings and straw [7]. Figure 5 shows a deep sawdust bedding system on a concrete floor base.

Due to the fact that it consists of very small particles, one of the main concerns of this type of bedding is that it is dusty; therefore, it may cause respiratory system disorder and eye irritation in horses [32]. Being a very fine material, its absorbing capacity is very high (215% water-holding capacity) and on average it also has an elevated dry matter of around 92% [7] compared to other beddings materials such as wood shavings and chips (dry matter content, 86%), straw pellets (dry matter content, 87%) [6] and wheat straw (dry matter content 89%) [25]. Provided the soiled sawdust is cleaned out regularly, this bedding type can be an excellent option to keep horses. From a human point of view, sawdust requires a higher workload as it needs to be mucked out frequently as bacteria and pathogens grow rapidly [33]. However, even if the small particles facilitate easier mucking out as they readily clump and are easy to separate, they could cause respiratory problems not only for horses but also for stable workers, especially during the mucking out process [33]. The airborne sawdust can be prevalent throughout the horse accommodation and settle on all exposed equipment and construction surfaces. Negatively, this requires ongoing cleaning which needs to ensure the fine particles are removed appropriately. The movement of air, horses and humans can all contribute to an influx of airborne sawdust particles. Minimising the horses’ exposure to airborne dust at times when new sawdust bedding is provided to the accommodation can assist with reducing respiratory irritation. Furthermore, the management of the accommodation’s ventilation system needs to be considered at all times to optimise sawdust extraction and minimise the overall level of airborne sawdust [33]. Sawdust can be considered to be environmentally friendly, particularly when it is a by-product of sustainable plantation species and wood processing and is used responsibly. Its biodegradability, compostability, and ability to enrich soil make it a valuable waste resource. However, improper disposal of sawdust can lead to environmental issues, particularly if it is discarded in large quantities in landfills. If not managed properly, it can contribute to methane emissions as it decomposes anaerobically in landfills [34].

#### 3.1.3. Wood Pellets

Wood pellets are a type of bedding material made from compressed dried wood or sawdust. They are usually packed and stored in bags. Unlike wood shavings, which vary in quality, wood pellets are consistent and reliable in their bedding characteristics. Wood pellets offer a dust-free and extremely absorbent solution as they expand when they absorb moisture such as urine. Pellets are safe for horses’ health because they are made entirely of untreated softwood without any additions [3]. The water-holding capacity of wood pellets varies depending on the wood types, environmental conditions, and pellet density. They can absorb approximately 30 to 50% of their weight in water. By keeping the environment dry, wood pellet bedding’s absorbency encourages healthy hooves and lowers the possibility of bacterial growth. Nevertheless, Nazarenko et al. [35] reported that wood pellets produced the highest concentration of airborne particulate matter (PM) of three size fractions compared to straw, shavings and STREUfex [35]. These results highlight the finding that wood pellets, even if dust-free, can cause additional airborne particles, particularly due to the activity of horses in their stable loose boxes [35]. In addition, horses were lying down and foraging significantly less on wood pellet bedding than on straw bedding [3]. For this reason, wood pellets appear to be less advantageous from a welfare point of view than other types of bedding [3].

Wood pellets are simple to handle, store, and discard. Christ et al. [3] conducted a study in which they examined wood pellets in a real-world setting at a horse stable with single stalls and compared them with wood shavings and wheat straw. In contrast to wood shavings (13.4 min) and straw (15.9 min), the authors discovered that wood pellets were the simplest to muck out, requiring less time and money, roughly 11 min per horse per day. Another positive characteristic of wood pellets is that they are more environmentally friendly as the organic matter easily decomposes when exposed to moisture [3].

### 3.2. Straw Bedding

#### 3.2.1. Straw

**Figure 6:**
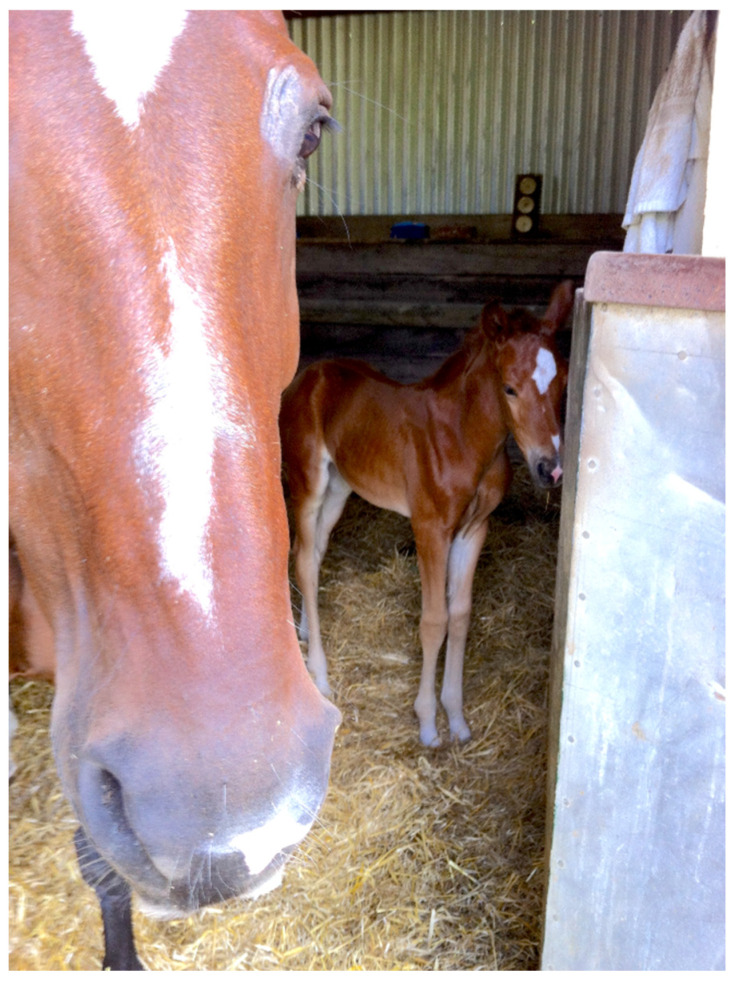
Deep straw bedding in a mare and foal box (Source: Lee’s photo).

Straw is the plant stalk that is removed from cereal grains like wheat, barley, oats, and rye. Like hay, the stalks are chopped, dried, and baled. Different from hay, this bedding material lacks seed heads and leaves [6]. Straw is the most common bedding material used for stabled horses worldwide [13]. Its popularity is related to the fact that it is widely available and generally an economical natural commodity. Straw has a 20–30 cm length, 89% dry matter content, and 321% water-holding capacity [6]. Straw is mainly used in individual loose boxes but, according to Briefer Freymond et al. and Christensen et al. [36,37], it may also be utilised as bedding for constructed shelters in paddocks. Horses benefit from the soft, insulating surface that straw offers [38], and it was reported that straw bedding protects horses from insect bite hypersensitivity (IBH), according to a study by Robin van den Boom and colleagues [39]. However, when choosing straw bedding, other selection criteria must be further considered as it can have an impact on horses’ health and welfare. If the quality of the straw is poor, it can be dusty or contain fungal spores causing or worsening respiratory diseases [25]. This aspect could be unpredictable as there could be a considerable difference in quality between different straw batches [6]. An experimental study by [40] discovered that rye straw generates the lowest gases such as ammonia (NH3), nitrous oxide, carbon dioxide, methane, and water vapor compared to wheat straw and wood shavings. This indicates rye straw is superior to wheat straw in optimising the air quality of the horse stable. However, it was reported that inflammatory airway disease (IAD) occurrence as a result of fungi increases in tracheal wash when horses were kept in mulch around trees or flower gardens or pasturelands bedded with straw [41]. Furthermore, if improperly harvested or stored, straw can be prone to mould [42], again contributing to the development of respiratory problems [32]. In addition, it was reported that inadequately managed straw can be a risk factor for the occurrence of hoof problems compared to other bedding materials [43]. Straw is palatable, especially when horses do not have other food available to eat [20]. For this reason, it can be used as a nutritional enrichment. In fact, according to the literature, horses may also play with straw and roll in it as well as eat it [44,45]. Stereotypic horses exhibit stereotyped behaviour less frequently when they are bedded on straw [46]. On the other hand, it is worth noticing that an excessive ingestion of straw could result in impaction colic [20]. In fact, horses were found to ingest straw for 8.1% of their time budget when they are stabled [47]. Another important consideration is that if straw is used in the resting areas of horses housed in groups, some horses could disturb the sleeping of others by trying to consume it [48]. Researchers have shown that straw bedding encourages resting/bedding-directed behaviour in horses and especially encourages lateral recumbency sleeping [6,25,47]. The latter is important to reach paradoxical sleep (i.e., REM sleep), essential to complete a sleep cycle and support the horse to rest well [49]. Furthermore, equine cyathostomines can develop into infective larvae on moist straw bedding. It is therefore possible for a horse in deep litter straw to become infected by ingesting the contaminated straw [50]. Therefore, the frequent replacement of straw bedding helps to reduce the cyathostomines infective larvae. Figure 6 shows deep straw bedding in a mare and foal box.

From a human perspective, straw bedding has both positive and negative aspects in regard to its use as a bedding. Among the most important advantages, which is also one of the reasons why straw is among the most used bedding, there is the low annual cost of the product, even though the price may vary depending on location, year, and season [44]. On the other hand, it requires a high daily demand for mucking out, increasing the amount of labour required and costs and the amount of faeces [44].

Environmentally, the use of straw bedding is considered to have a positive impact, as it can be included in an ecological cycle [51]. There are new options for processing used straw as an energy resource [52]. One of these is anaerobic digestion, which offers additional benefits, such as lower carbon emissions and a reduction in methane production [13,53]. A study conducted by Boske and colleagues showed straw mixed with horse manure has the highest potential for methane production compared with other bedding material/manure mixes such as hemp, flax and wood chips [54]. Wheat straw has the highest score in biochemical methane potential (BMP) compared to other bedding materials such as flax, hemp and wood chips [52]. This indicates that straw bedding mixed with horse manure has great potential to be used as an alternative energy source. In addition, a study by Komar et al. [55] indicated that simple aerobic digestion greatly reduces the manure waste volume, and the material could be used as a natural fertiliser in pasture-based systems. The straw-based material was found to be suited best for organic composting. This study is an additional example demonstrating how straw bedding materials can be easily included in the ecological cycle without a negative environmental impact.

#### 3.2.2. Straw Pellets

Straw pellets are a commercially available bedding material made of compressed straw [6]. However, they are less common due to their reduced availability than other types of bedding [3]. They usually have 87% dry matter content and 419% water-holding capacity [3]. A study by Fleming et al. [56] found that straw pellets had the lowest particle generation and the highest water-binding capacity when compared to other bedding materials such as straw, shavings and shredded paper. Furthermore, another study by Fleming et al. [57] discovered that straw pellets had the lowest ammonia concentration compared with other bedding material such as straw, wood shavings and shredded paper. As a result, the researchers recommended straw pellets as a good alternative bedding to improve the air quality in a horse stable, as well as in relation to ammonia binding and ammonia transformation within the bedding material. However, it was highlighted that the high substrate temperatures found in straw pellets may also encourage the growth of pathogenic microorganisms that could have a negative health impact on horses [56,57]. Additionally, Monki and colleagues [10] discovered that healthy horses kept on straw pellets had a greater proportion of neutrophils in their lower airways than horses kept on peat bedding. Another disadvantage of straw pellet bedding that the literature highlighted is the apparent reduced comfort level for horses when compared to straw and wood shavings [38]. In this study, the horses demonstrated fewer lying behaviours while managed on this bedding type.

From a human perspective, Monki and colleagues [10] state that pellets can be considered more practical in the management of horse bedding as smaller volumes of replacement material are needed and mucking out may be less time-consuming and arduous when compared to the traditional straw and wood shaving options. No research was identified that evaluated the health consequences of the pathogenic microorganisms identified [56] on stable workers and visitors. Further studies are required to fully investigate the human health and welfare implications of utilizing straw pellet bedding.

Environmentally, straw pellets are ecologically sustainable as they are plant-made and can be readily disposed of. The organic matter is biodegradable and will decompose into a material that can be beneficial to the environment and used in pasture management [6]. In addition, straw pellets could be used to produce methane and serve as an alternative energy source as they produce the highest methane content through anaerobic digestion compared with other bedding materials such as flax and woody materials [58].

### 3.3. Peat Moss Bedding

Peat moss is usually made of partially decomposed sphagnum moss [59] and is a natural and popular option for bedding due to its remarkable absorbency (14.7 L of peat needed to hold ten litres of water) [59] and soft texture [23]. The first peat moss industry producing peat for horse bedding opened in the Netherlands at the beginning of 1900. At that time, peat was in great demand both for city stables and for cavalry horses [60]. Nowadays, peat moss is used especially in Northern Europe and is quite common in Finland and the other countries of the Baltic area [23,31].

The absorbency and cushion make it an excellent choice for senior horses and those with allergies, heaves, or inflammatory airway disease. Compared to wood shavings and straw pellets [10], it has been demonstrated to lessen respiratory inflammation in horses. Specifically, the tracheal wash and bronchoalveolar lavage fluid contained fewer neutrophils in horses who were managed on peat [23]. Additionally, peat moss has natural antimicrobial properties, contributing to a clean and hygienic environment. However, a study found that hoof horns’ moisture content was greater in horses stabled with peat bedding compared to wood shavings, highlighting the possibility of hoof problems [1]. Furthermore, it was discovered that horses sleeping on peat moss had more undesirable behaviours, such as bar biting and lignophagia [2]. Lastly, it might not be the greatest option for white or grey horses because of its extremely dark natural colour, which could potentially discolour a lighter horse’s coat and tail.

From a human perspective, peat moss is easier to handle than straw [60] but, currently, it can be costly and difficult to source in certain locations. It is easy to muck out and usually delivered in bags making it easy to store [61]. Peat moss is environmentally friendly, and ecological disposal is easy due to its easily decomposing nature [59].

### 3.4. Rubber Matting

**Figure 7:**
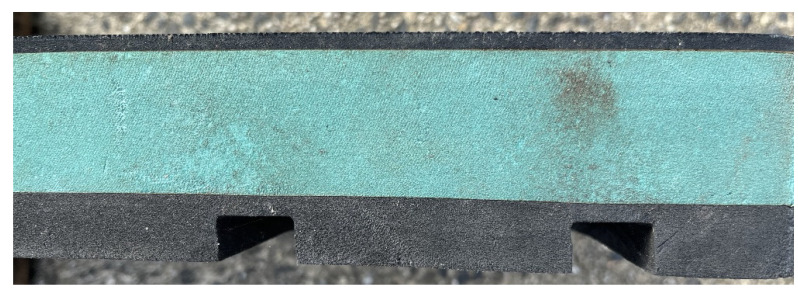
Detail section of a high-end rubber profile which combines various rubber types to provide comfort (green section), durability (hardwearing top section) and the channel profile on the underside for drainage and further cushioning (Source: Lee’s photo).

Rubber mats are a popular bedding alternative to natural-based bedding systems. The industry has launched rubber mats on the market primarily as a durable, non-palatable solution for horses kept in groups and to reduce the need for deep fibre-based bedding systems and their associated labour-intensive management. The main question that researchers are investigating is whether they can be considered an adequate bedding type or just a good type of flooring. This is because among the fundamental characteristics of bedding is absorption and in the case of rubber matting this characteristic is totally absent, so its water-binding capacity is zero. There are notable differences in the characteristics and functionality of various rubber mattings. This encompasses profile, hardness, traction, slide, durability, density, and thickness. For instance, hefty, specially designed vulcanized rubber mats that are 12 mm thick and compression-fitted into a box can offer lifetime traction, high insulation, and a metal-free guarantee. Figure 7 shows a detail section of a high-end rubber profile which combines various rubber types to provide comfort (green section), durability (hardwearing top section) and the channel profile on the underside for drainage and further cushioning. Other mats, including new and/or reclaimed conveyor belting from the manufacturing industries, can be economical options and very durable with their various nylon and steel types available. However, these profiles can be extremely hard underfoot for both people and horses and offer minimal comfort from the concrete flooring base beneath. Negatively, they can offer little traction and be slippery for shod horses, particularly when wet. There are also a number of rubber-based matting systems available on the market that are mattress types. These generally consist of a multi-layered system including a high-density top cover with low-density rubbers and rubber crumb, arranged in a cell arrangement. Although each mattress brand has a unique profile and physical attributes, the main objective is to provide comfort for the horses. They can be exceedingly robust and absorb significantly more shock than solid rubber mats and offer the horse greater comfort and greater cushioning than solid rubber matting.

Some rubber mat profiles have been found to be uncomfortable for horses, with horses having shorter lying times where rubber was installed in their accommodation [51]. In a study by Burla et al. [62], the behaviour of group-housed horses was tested when allowed access to different types of ground, namely firm ground, rubber mats and litter. It was found that horses lay for longer periods on rubber mats when they were covered with some level of litter. The same results were also found by Baumgartner et al. even if they used a different type of rubber mat (i.e., HIT Horsebed Premium) [48]. The authors concluded that horses could accept rubber mats as bedding only if minimally covered with other natural bedding materials and they were of adequate/appropriate cushioning for comfort.

Rubber mats with a minimum amount of shavings promote a good stable thermal condition if appropriate ventilation is given and manure is removed multiple times a day [63]. Especially during heavy rain and snowfall, shavings on rubber mats ensure a dry and nonslip lying surface. Under these conditions, rubber mats can be considered suitable for horses in terms of animal welfare for group-housed horses [48]. In addition to providing a level of temperature insulation, different rubber mats provide varying sound absorption properties. This can be advantageous for both horses and humans where stables are in close proximity to other buildings and adjacent properties and the noise from the horses, people, and their associated activities can be tempered by the rubber surface. Studies have indicated that soft rubber mat systems with a reduced bedding depth also save significant labour time (up to 28% less work) when mucking out [63], which is a clear benefit for stable workers. Environmentally, there are fewer benefits in rubber matting when compared to the other bedding alternatives reviewed as rubber mats are inorganic and therefore they do not decompose [64]. However, rubber matting manufactured from recycled rubber products such as discarded vehicle tyres can reduce landfill waste and can therefore be considered as having some environmentally friendly elements. Their production is less resource-intensive than creating new rubber, and they provide a safer, non-toxic alternative to other synthetic materials. However, their environmental friendliness can depend on factors such as the presence of chemical additives, the energy used in manufacturing, and the disposal methods at the end of their lifecycle. Overall, when used appropriately, recycled rubber mats can be a sustainable and durable choice for many horse accommodation applications [65].

### 3.5. Paper Beddings

Shredded paper is one of the bedding materials made of paper, and it has become a novel bedding option on the current market. Paper products used as a bedding material are less common, but they has many benefits, such as superior moisture management and absorption. Paper bedding types have a high water binding capacity (i.e., 400%). According to a study performed by Ward and colleagues [66], the water-holding capacity of paper used for bedding was high compared to wheat straw (400 vs. 200%). Shredded paper is ideal for horses with allergies or respiratory sensitivities since it effectively absorbs moisture and is dust-free. Tanner and colleagues [67] compared phonebook paper bedding to other common bedding types (i.e., straw and sawdust) to identify the possible differences in the presence of airborne Gram-negative bacteria, fungi and endotoxins. They concluded that no statistical differences were found between these three bedding types and that phonebook paper may be an acceptable bedding in terms of microbial contamination [67]. However, it does require more frequent mucking out and is susceptible to mould if not cleaned regularly. This effectively creates a greater workload for stable staff and incurs a greater cost in terms of time and money [31,68].

As for wood and straw bedding types, paper can be processed and compressed to form pellets. This type of bedding was tested by Ward et al. and they concluded that considering its chemical, physical and environmentally sensitive properties this bedding material has a high potential for market success [66]. Paper bedding is an environmentally friendly option because it can be recycled or composted and is a good environmentally responsible substitute for horse bedding [31,68].

### 3.6. Fibre-Based Beddings

Flax and hemp are bedding materials made of natural fibres. Flax fibre crops grow primarily in Northern Europe. A byproduct of flax fibre processing is the “shive”, the woody core of the stalk. As a horse bedding, it is highly absorbent, and a small amount is required to perform this task [69]. In the study by Holzhauer and colleagues, they described flax as offering better protection for hooves compared to other bedding materials such as straw [43]. Flax bedding is a vegetable product, free of dust and pests. Flax is usually composed of about 53% cellulose, 21% hemicellulose, 24% lignin, and 2% ash making up flax shives in general [69]. An experimental study by Borhan et al. [70] compared flax and pine wood shaving bedding materials regarding their water-binding capacity and gas concentration. The study determined that flax is an advantageous bedding material with its superior binding capacity, being easier to clean and having a lower dust level in comparison to pine wood shavings. One of the disadvantages of flax bedding is that it can be slightly slippery when first laid down. Additionally, it raises the risk of colic; according to a retrospective study conducted in Belgium, where 11.3% of colic cases were stabled on flax bedding, a multivariable logistic regression model showed that horses stabled on flax shives suffered an impact on their ileum roughly three times more frequently than horses stabled on straw [69]. From the human perspective, flax bedding is usually considered healthy and convenient, as it can be purchased in bags and is easy to transport. Finally, flax bedding is an environmentally friendly product since it is a vegetable-based product and once the manure is removed, the used flax is completely compostable, given its neutral pH value [9].

Hemp is another relatively new fibre-based bedding material made of fibres of hemp vegetation. The soft, middle portion of the hemp stem is used to make hemp litter [71]. It is extremely absorbent (up to four times its original weight), and 22.5 L of hemp is needed to hold ten litres of water [59] and is mostly composed of cellulose and lignin [71]. Silica, a chemical that occurs naturally in sand and flints, is also abundant in hemp stalks [71]. Equine management considers it favourably due to it being easy to distribute and store. Further positives in utilising hemp bedding include its low dust content and a high ability to reduce airborne odors. One of the disadvantages of hemp is that it should not be used in racehorse stabling accommodation when horses are treated with non-psychoactive cannabinoids cannabidiol (CBD) as hemp absorbs it for a very long period. As cannabidiolic acids (CBDAs) are banned substances and horses can consume the hemp bedding, these substances can be present and detected in their blood for an indefinitive period of time [72]. It is worth also highlighting that one study determined that hemp and flax litter was the bedding type with the highest concentrations of ammonia (around 8 ppm), which is a high risk for both horse and human health [9]. However, in consideration of the human workload, hemp bedding is easy to muck out and reduces both the labour effort and associated labour costs.

Environmentally, hemp is biodegradable and eco-friendly [9]. As a plant species, hemp is naturally resistant to most pests so requires minimal pesticides and herbicides in its crop production. This significantly reduces its chemical impact on the natural environment. Furthermore, hemp has a short growth cycle and requires minimal water which is also positive for the environment [71]. Only a few studies have been conducted on this type of bedding, so the information available to review is limited.

### 3.7. Rice Hulls

Rice hulls are a natural type of bedding, as they are a byproduct of rice processing. They are frequently utilised in places that produce rice as a food source [9]. Compared to wood shavings, rice hulls are lighter and less dusty. Rice hulls have an adequate ammonia and moisture value. Similar to rubber matting, they should only be used as bedding in stalls with adequate drainage or in conjunction with other bedding types as they are not very absorbent. Rice hulls constitute a sustainable solution for horse bedding [9]. They are generally considered environmentally friendly, particularly when used as a byproduct of rice farming, due to their biodegradability, renewable nature, and potential for recycling and repurposing.

### 3.8. Sand Beddings

**Figure 8:**
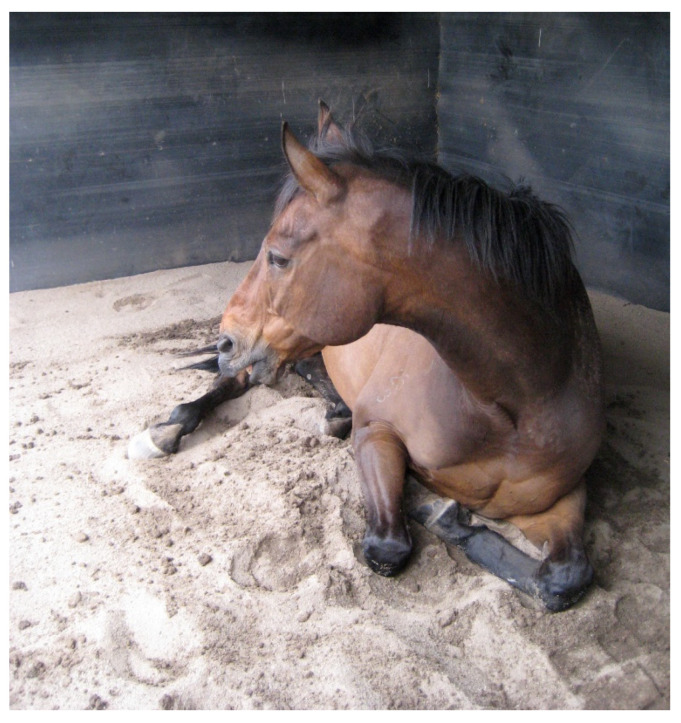
Sand bedding can be effective for older horses that tend to lie down for long periods of time as it provides good support and comfort (Source: Lee’s photo).

Sand is not a common type of bedding. However, it is recommended in some pathological situations, such as horses with laminitis or those who tend to lie down for longer periods due to various orthopaedic problems, as it can be quite comfortable [73] and horses rest on it longer than on other options [48]. Sand bedding could also be used to reduce the development of house flies in areas where it is a problem for horses in stables [74]. Figure 8 shows sand bedding can be effective for older horses that tend to lie down for long periods of time as it provides good support and comfort.

Sand has been suggested as an option for use in group housing accommodation as it is not generally palatable [48]. However, one of the main disadvantages of this type of bedding is that horses can ingest sand while eating hay on the ground, which can lead to sand colic syndrome [75]. Sand bedding has a moderate absorption capacity (between 130% and 200%, depending on the type of sand), which can assist in keeping the accommodation dry and comfortable for the horses [64].

From a human perspective, sand bedding can be easily cleaned and is reasonably priced, but some bacteria may persist over time making the environment non-hygienic for both horses and humans. Environmentally, sand is difficult to dispose of as it is not organic; therefore, sand bedding is not an ecologically sustainable choice [64].

### 3.9. Coconut and Coffee Beddings

Coconut bedding is a natural and ecological product that is soft, homogeneous and easy to distribute. It is considered to be of a pleasant appearance and due to its high absorption capacity, the litter is always very dry on the surface. Coconut bedding provides the total elimination of odours, which is of benefit to both horses and humans. Significantly, coconut bedding maintains optimal hygienic conditions and prevents the onset of serious diseases such as [9]. Coconut bedding produced the lowest ammonia concentration values (1 ppm) in a study comparing it to other bedding materials (such as flax, rice, hemp, and wood shavings) and it is characterised by a total absence of dust [9]. These characteristics are beneficial for both horses’ and stable workers’ respiratory health and as it is not palatable for horses, there is no risk of colic. As a further positive attribute, this material is considered comfortable, as a study found that horses spend more time in lateral recumbency when accommodated on coconut bedding compared to sawdust [76].

Coconut bedding material is considered environmentally friendly because it reduces the amount of manure to be disposed of, which is still of premium quality and acceptable for agronomic uses such as a simple fertiliser or composting [9].

Coffee husks are another type of bedding especially used in Latin American countries that produce large amounts of coffee, such as Brazil. Coffee husks are the main waste product from coffee grain processing. They are rich in organic matter and contain tannins, polyphenols and caffeine. Despite being an excellent reuse of a waste product and eco-friendly, it has been reported that ingestion of this bedding type can cause intoxication in horses and other animals. In particular, the ingested caffeine causes hyperexcitability resulting in tremors, profuse sweating, involuntary tongue and lip movements and an increased heart and respiratory rate. The symptoms usually last up to 24 h from when the animal stops ingesting the substance. Therefore, this type of bedding should not be used in any horse accommodation [77,78].

### 3.10. Comparison Beddings Using a One Welfare Approach

Table 2 presents a summary of the common bedding materials, and their advantages and disadvantages in terms of horse and human welfare, and the environment.

When considering horse welfare, it is crucial to evaluate how different bedding materials impact the horse’s comfort, health and behaviour. In summation/in regard/in reference to/for horses: straw is widely used as it promotes lateral recumbency, which supports natural resting behaviour [25]. However, straw poses risks such as ingestion, which can lead to impaction colic [20], and its lower absorbency can contribute to ammonia build-up if not properly managed [35]. Even if dried, straw still can carry high ammonia which makes it difficult to be reused [79]. Wood shavings are highly absorbent and help control moisture and bacterial growth, benefiting hoof and respiratory health when dust levels are minimised [6]. However, dustier shavings can pose respiratory risks for horses [22]. Wood pellets provide superior moisture absorption and create a dry environment, but their higher ammonia concentrations can negatively affect respiratory health [3]. Peat moss, with its excellent absorbency and soft texture, is ideal for horses with joint or respiratory issues [23]. However, it has been linked to undesirable behaviours like bar biting [2]. Rubber mats provide a non-slip surface that reduces the risk of falls and offers good support for horses, particularly those with orthopaedic issues [51]. However, they do not absorb urine, which generally necessitates the use of additional, albeit reduced bedding litter to manage moisture and encourage resting behaviour. Therefore, rubber mats, when combined with suitable bedding, could be an excellent choice depending on the horse’s individual health and behavioural needs.

From a human perspective, straw is cost-effective and easy to source, but its low absorbency requires more frequent mucking [35], and the dust generated can irritate stable workers’ respiratory systems [42]. Wood shavings, while reducing mucking frequency and time, are more expensive and may release dust that aggravates respiratory issues for stable workers [22]. Wood pellets also reduce mucking time, but their higher ammonia concentrations can affect air quality, posing health risks to workers too [3]. Rubber matting is durable and reduces the frequency of mucking out, but it comes with a high upfront cost and can trap dust, potentially decreasing air quality [68]. Sawdust is inexpensive and absorbent, but its high dust content can be harmful to workers’ respiratory health [33], whereas shredded paper is dust-free but requires more frequent mucking out and can be costly [68]. Overall, while bedding materials that reduce worker health risks and mucking out time may be more expensive initially, considering the long-term costs of health issues and labour reduction, dust-reduced or dust-free wood shavings may be the optimal choice for everyone involved in stable management.

In terms of environmental impact, straw is biodegradable and offers environmental benefits when managed properly, especially if sourced sustainably [51]. However, its production and transportation contribute to environmental degradation, particularly when not sourced responsibly. Wood shavings are biodegradable and can be sourced sustainably from certified forests, but they still carry an environmental footprint related to their production and transport [6]. Wood pellets, made from sawdust and wood by-products, have a relatively low environmental impact in terms of waste usage, though their production requires significant energy, and their higher ammonia emissions can reduce their overall sustainability [3]. Peat moss raises environmental concerns due to its extraction process, which leads to carbon emissions and habitat disruption [61]. Rubber matting is durable and reduces the need for frequent bedding replacement, offering long-term sustainability, but its production and disposal can have significant environmental costs [68]. Sawdust is biodegradable but can contribute to particulate pollution if not managed properly [33]. Finally, shredded paper is an eco-friendly choice due to its recycling potential, but its production requires considerable resources, and its mould susceptibility can affect its sustainability if not managed correctly [68]. Overall, when considering environmental impact, it is important to choose biodegradable bedding materials that are sustainably sourced and disposed of correctly, minimising waste and harmful emissions. A study by Woodward et al. [80] showed that different bedding material used in horse stables has a different level of phosphorus and nitrogen concentrations. The study compared the environmental load of phosphorus and nitrogen in six bedding materials: peat moss, wood shavings (WSs), wood pellets, straw, chopped straw, and corn cob hulls. The environmental waste load of phosphorus and nitrogen was different in the six bedding materials where peat moss has the highest concentration of both phosphorus and nitrogen. The researchers concluded that in order to mitigate the environmental effects of horse facilities, the horse industry should focus on lowering the quantity of bedding removed with waste, putting in place suitable waste treatment systems, and minimising the surplus nutrients that horses excrete through adequate nutrition [80].

## 4. Discussion

Using the One Welfare approach, this review examines the most common bedding materials used in horse stables, focusing on their impacts on the well-being of both horses and humans, as well as their management advantages and disadvantages and known environmental effects. Stable managers must consider a variety of factors when selecting bedding, including sourcing, capital investment, ongoing costs, delivery, storage, installation, maintenance, and disposal [13]. This study aims to support and give evidence for this decision-making process. The evidence was gathered through a systematic literature review. The review identifies the gaps in knowledge and recommends areas for further research. While more extensive studies and reviews have been conducted on bedding materials for dairy cows [64,81], pigs [82] and poultry [82], the equine industry has lagged behind, especially regarding the environmental impact of these materials. Given the high number of horses worldwide, many of which are kept by small farmers and managed by various stable managers and owners, there is a pressing need for education on selecting the most suitable bedding to optimise not only horse welfare but also the well-being of stable workers and consider the environmental impacts.

The horse is the primary interested party in the bedding material choice, with bedding having a major impact on the horse’s health and welfare. Among other things, bedding influences comfort around resting, lying behaviour, and hooves and respiratory tract health [7]. It plays a crucial role in influencing air quality, directly impacting the respiratory health of horses. Poor air quality is a significant factor contributing to various equine respiratory conditions, such as equine asthma, recurrent airway obstruction (RAO), chronic obstructive pulmonary disease (COPD), and inflammatory airway disease (IAD) [83,84]. Horses suffering from these conditions should be managed individually by selecting the bedding materials which may minimise their respiratory disorders [7]. Different methods could be used to reduce the concentrations of inhalable and respirable airborne particles in horse stables such as oil spraying and oil impregnation in the bedding material [85]. It is important to consider the bedding’s softness, texture, fragrance and insulating qualities in the choice of material. It is generally advised to use 15 to 20 cm of bedding [7], but the depth also depends on the material type used. When determining the depth of the bedding, the type of flooring base should be taken into consideration; otherwise, none of the bedding materials described may be sufficiently comfortable. However, the majority of the materials and methods in the studies cited did not include any information regarding the depth or quantity of bedding tested, making it difficult to suggest the ideal depth. The bedding provided should be deep enough to optimise a soft and comfortable surface for the horse to lay down and sleep. Although horses are among the mammals that require the fewest hours of sleep (about 3 h/day), they still need a comfortable sleep phase in lateral or sternal decubitus to reach the REM phase of sleep [86]. In addition, it is a common practice to place more bedding material along the sides of the loose box to reduce the possibility of the horse getting cast while rolling. This procedure is often practised but there is no scientific evidence on its effectiveness. There are other factors, including the size and shape of the box and the types of wall panels, that can affect the possibility of a horse getting cast while rolling, being unable to get up or REM sleep [33,87].

The bedding material used in the horse stable can alter the feeding behaviours of horses especially when they are housed on non-edible bedding material. According to the study by Baumgartner et al. [88], the horses’ ethological feeding needs are not satisfied on non-edible bedding. In the study, wood shavings caused longer fasting periods compared to straw where there was no other ad libitum roughage feed source available for the horses. Therefore, only edible bedding materials could be considered welfare-friendly where horses have limited access to roughage and specific feeding management in horses kept on non-edible bedding material should be engaged to limit any welfare impairment [88].

Additionally, absorbency is an important characteristic to consider in preventing disorders to the horses’ hooves and respiratory systems. However, the impacts on the hoof and respiratory health are not the only health aspects to consider, as some beddings may increase the horses’ exposure to toxins, mould and bacteria or may be ingested, increasing the risk of colic [9].

The bedding material used in horse stables not only influences the horses’ welfare but also has an impact on the welfare of the personnel working in the horse industry, such as horse caretakers, farriers, veterinarians and visitors [1]. People working in the horse stables may be exposed to different toxic substances and organisms present in the bedding material, leading them to respiratory disorders [1]. The dust particles can carry bacterial endotoxins and fungi, presenting respiratory hazards for humans working and visiting horse stables [89]. Furthermore, from a management and business perspective the initial cost, the longevity and how often bedding needs to be replaced or mucked out, and whether it is easy to muck out are factors that should also be considered in the bedding selection.

The handling of horse stable bedding waste in combination with solid manure is becoming a more significant issue for equine operations because of the accompanying expenses and environmental concerns. The production, use and disposal of bedding material also has an impact on the environment. Hence, considering whether it is biodegradable or not is important from a sustainability and health perspective [9]. Additionally, horse excrement has the potential to be converted into valuable products or energy [90]. This necessitates a thorough understanding of the material’s properties, knowledge of the quantities that are available, and an evaluation of appropriate treatment technologies [13]. This may include a rigorous investigation for utilising the bedding waste and manure as a renewable energy resource, and/or a natural fertiliser. Some of the selected manure management processes include composting processes, anaerobic digestion and combustion which help in reducing the impact of bedding material waste [80,90,91].

Our review has some limitations which need to be considered when interpreting the results presented. First of all, the literature search was limited to the Scopus and Web of Science databases. This may have created a bias as there are some criteria to be listed in these portals. Moreover, in the search, some filters, in particular the language (English), the scientific areas, the type of documents, and the years of publication, were applied, and this could have limited the number of records retrieved. Finally, the selection of keywords, which was discussed among the authors, may have omitted some aspects, such as the environmental impact, as this is less evident. Notwithstanding these limitations, we have endeavoured to summarise the common bedding materials, and their advantages and disadvantages regarding horse welfare, humans, and the environment. Based on the currently available literature, it is difficult to rank the types of bedding and to determine which bedding material is the best due to the fact that extensive studies that consider the various facets, including horse welfare, human and environmental impact, are lacking. There are no studies with a comprehensive comparison, where all the listed beddings were evaluated. Indeed, almost all the studies that were listed compared the impact of two to four different types of bedding on a single criterion, such as resting behaviour, air quality, or tracheal wash neutrophils of horses. Consequently, studies using a multidisciplinary approach are recommended to be able to identify the optimal bedding material to use in equine establishments to optimise and enhance not only horse welfare but also human well-being and have a positive environmental impact. Moreover, from the authors’ point of view, the bedding choice must consider the individual needs of the specific situation, namely the horse and horse worker’s health status, the type of husbandry system and the context (including the country, locality, authorities, climate, etc.) where the horse accommodation is located.

## 5. Conclusions

This literature review presented the documented advantages and disadvantages of several bedding materials used in horse stables regarding equine and human well-being and the environmental impact. Our findings provide useful information for equine industry members in supporting their decision-making process when it comes to the selection of bedding systems for any stabling facility, which in the authors’ view should ultimately be tailored to each individual horse, and husbandry system. There are different types of bedding for horses, of which straw and wood shavings are the most used and studied. Traditionally, straw is the most popular and prevalent bedding material which is generally readily available to horse operations with access to foliage-based crop farming. As it is edible, though, it can cause impaction colic, and in group housing, horses may annoy other horses who are attempting to eat it. It can also be a source of airborne contaminants which can negatively affect the horse’s well-being. The second most popular bedding is wood shavings with its superior absorbency. Since there is a wide variety of wood shavings available, it is crucial that the specific bedding material sourced is de-dusted, and dehydrated. Although manufacturer literature exists on alternative beddings, there are very few scientific studies on their effects on the horses’ and, humans’ well-being and the environmental impact. Further studies regarding the advantages and disadvantages of each bedding material exploring horse and human welfare and the environmental impact using a multidisciplinary approach are therefore recommended to rank the bedding and suggest the optimal type/s using the One Welfare approach.

## Figures and Tables

**Table 1:** Exclusion and inclusion criteria applied to screened records.

Inclusions	Exclusions
- Studies on the impact of bedding on horse health and welfare - Studies on the impact of bedding on human health and welfare - Studies on the impact of bedding on the environment in terms of recycling or producing energy	- Studies using the word bedding/flooring materials and methods but with a different aim related to another equine science area (i.e., clinical studies, nutrition, microbiology, parasitology, genetics) - Studies on humans, horses or environment but not describing the effects of bedding, or having ‘bed’ but not bedding (i.e., wrong topics) - Studies describing some historical aspects (i.e., Archaeology) - Studies examining the bedding as a fertiliser and assessing new technologies for the production of bedding and other by-products without describing any effects/implications on/for horses, humans and the environment (i.e., Agri-Technology) - Studies on a different species (wrong species) - Studies focusing on the effects of a specific diet in equidae (i.e., nutrition)

**Table 2:** Summary of the common bedding materials including their advantages and disadvantages for horse and human welfare and the environment.

Bedding Material	Advantages	Disadvantages
Wood shavings and chips	Horses - Shavings and chips can support healthy hooves [6]. - They can reduce bacterial growth in the bedding material and skin irritations [6,24]. - Some wood shavings have fewer allergens [6]. - The dust-reduced variety is beneficial to horses with respiratory conditions - The cushion provided by shavings and chips can promote longer recumbent resting times for horses in comparison to straw [6]. Humans - The mucking-out time for stable workers can be reduced due to its manageability and superior absorbency when compared to straw. [26] - High-quality wood shavings can be durable [26]. Environment - Shavings and chips can be environmentally friendly if sourced from sustainable wood harvests and processes. - Wood shavings and chips are biodegradable and provided it is untreated, the waste is biodegradable and can be disposed of in landfill and/or reused as mulch or compost. - Wood shavings manure can be used as a compost [29].	Horses - Some wood species can be toxic to horses such as black walnut, which is poisonous to horses and if ingested, can cause acute laminitis [21]. - It can be dusty and can contribute to the risk of respiratory disease [22]. - It can provide a higher risk of inflammatory airway disease (IAD) [22]. - Due to its reduced insulative qualities, it can provide lower warmth in comparison to other bedding materials [22]. - It can support high ammonia concentrations [1]. Humans - High-quality wood shavings can be more expensive to purchase than straw [6,26]. - If not dust-reduced, this bedding material can be deterimental to the welfare of people with respiratory conditions. - The dust can be prevalent throughout the horse accommodation and require the ongoing cleaning of equipment and the construction. Cleaning and the accommodation’s ventilation design need to consider the removal of dust and air movement control.
Sawdust	Horses - This bedding material can provide a dry environment for the horses provided the accommodation is cleaned out frequently [7]. Humans - It can be easier to maintain with a reduced muck-out time in comparison to other deep bedding systems due to its high absorbency and clumping characteristics [7]. - Sawdust is generally less expensive to purchase than wood shavings and straw [7]. Environment - Sustainably sourced sawdust is categorized as environmentally friendly [34] and provided the sawdust was untreated, the waste is biodegradable and can be disposed of in landfill and/or reused as mulch or compost.	Horses - Sawdust is inherently dusty and can cause respiratory and eye issues [32]. - It can readily support bacterial growth and pathogens and requires frequent mucking out to minimise contamination build-up [33]. Humans - Sawdust can be detrimental to humans’ respiratory health [33], particularly for people with respiratory conditions. - The ongoing exposure to fine sawdust particles can be difficult to manage and minimise. Environment - The improper disposal of large quantities of used sawdust bedding can lead to methane emissions.
Wood pellets	Horses - This is generally considered a safe bedding option in terms of the horses’ welfare [3] by providing a dry environment, supporting good hoof health and lowering the possibility of bacterial growth within the bedding material. - The wood pellet bedding is consistent in its characteristics due to the control in its manufacture process. - It can be dust-free and highly absorbent [3]. Humans - Packaged in bags, they are easy to handle and store. - With their great ability to absorb moisture, muck out labour and time can be reduced [3]. - With overall less waste, there can be a reduction in disposal labour and cost. Environment - The biodegradable wood pellet bedding is a natural-based product and considered to be environmentally friendly [3]. - It can decompose quicker than some other bedding options without reducing the quality of the soil. - Due to its high absorbency, there is less material required and less waste to be disposed of.	Horses - Wood pellets can reduce the horses’ lying and foraging behaviour in comparison to straw [3]. - They have been reported to have a high concentration of airborne particulate matter compared to other types of bedding which can affect the respiratory health of the horses [35]. - They can support higher ammonia concentrations [3]. Humans - The potentially high concentration of particulate matter can cause respiratory concern for humans in the accommodation. - Ammonia concentration can be a respiratory irritant and health hazard. Environment - Provided the source and manufacturing process for the wood material was sustainable and eco-friendly, there were no disadvantages for the environment reported in the literature for wood pellets.
Straw	Horses - Straw is a comfortable, insulative bedding option and these characteristics promote horses’ lateral recumbency [25]. - Given straw’s potential for providing environment enrichment, including its palatability, there may be reduced stereotypic behaviours in horses bedded on straw [46]. - Straw protects horses from insect bite hypersensitivity (IBH) [39]. Humans - Soiled straw can be easily disposed of after use [38]. - Straw is considered to be an inexpensive bedding option [44] where it is readily available. Environment - Straw is biodegradable and environmentally friendly [51]. - Straw manure can be used as a natural fertilizer after aerobic digestion [55]. - Straw waste has the potential to be utilised as a new energy resource [13,52,53,54].	Horses - Straw can be dusty [42] and as a natural bedding option, it can be difficult to control the quality and consistency. - It is prone to fungi growth, mould and rot where moisture is present, leading to respiratory problems [32]. - Excessive ingestion of the straw may cause impaction colic [20]. - Straw bedding can cause hoof problems if not properly managed [43]. - Horses in group accommodation can be disturbed by other horses eating the straw bedding [48]. Humans - It requires higher labour intensity with daily mucking out more difficult in comparison to other highly absorbent bedding materials. - The amount of faeces and soiled straw waste to be disposed of is increased [44].
Straw pellets	Horses - Straw pellet bedding has the lowest particle generation and highest water binding capacity. This can make for better quality air and dryer bedding overall [56]. Humans - Straw pellets can be easily mucked-out in comparison to straw bedding due to their high absorbency and clumping. - Soiled bedding can be easily disposed of [6]. Environment - Straw pellet waste is biodegradable and ecologically sustainable [6]. - Straw pellets manure can be used to produce methane which serves as an alternative energy source [58].	Horses - Straw pellets may contribute to respiratory problems in horses [10]. - They are reported to be less comfortable than straw and wood shavings and do not promote horses’ lying behaviours, including recumbent sleep [38]. - The straw may encourage the growth of pathogenic microorganisms [57]. Humans - This bedding may negatively affect human health given the reported equine respiratory concerns and potential production of pathogenic microorganisms [10,56]. Environment - Straw pellet bedding has no reported environmental disadvantages in the literature reviewed.
Peat moss	Horses - Peat moss bedding can lower the possibility of respiratory inflammation in horses [10]. - It has remarkable absorbency which assists in keeping the environment dry and a soft texture [10] which can promote horses’ lying behaviour. - It has natural antimicrobial properties for horses [1]. - This bedding is an excellent choice for older horses due to the properties detailed above. Humans - Peat moss is light and easy to handle and store in bags. - Its high absorbency reduces the labour effort in mucking out. Environment - As a natural bedding option, it is fully biodegradable and eco-friendly.	Horses - The use of peat moss bedding has been reported to encourage undesirable behaviours like stable bar biting and lignophagia [2]. - It is not advised to be used where it is in contact with light-coloured horses due to the peat moss dark colour and potential to stain the horses’ hair [2]. Humans - Peat moss is costly and can be difficult to source [23,31]. - Due to its tendency to stain light-coloured horses, extra workload is required to clean and maintain horses bedded on peat moss. Environment - The peat extraction process of the naturally occurring resource negatively affects the ecosystem [61].
Rubber matting	Horses - Rubber can reduce reduces horses slipping [51]. - Rubber can provide an insulative, durable and constant cover to the flooring base, limiting potential injuries to horses caused by exposure to the floor base. Humans - Rubber is easy to clean, [51] and to optimise biosecurity. - Rubber can reduce the long-term cost of bedding with its lesser requirement for litter material and labour. - A variety of rubber matting systems is readily available and choice can be specific to the context and capital investment. Environment - Some matting products recycle the waste rubber from the vehicle tyre industry.	Horses - As rubber does not absorb urine [51] it can splash the horses‘ legs. - Rubber can limit the horses‘ comfort level and discourage lying behaviours [51]. - Rubber needs frequent cleaning [68]. - Rubber matting can accommodate dust and airborne particles [68]. Humans - Some rubber matting systems are heavy to install and consequently to remove for cleaning beneath. - Depending on the rubber product selected, the capital investment can be significant and limit the choice and quality. Environment - Rubber may not be environmentally friendly depending on its manufacturing, and disposal methods.
Paper bedding types	Horses - Paper bedding is ideal for horses with allergies or respiratory sensitivities. - It can be three times more absorbent than wood shavings [66] - and offer superior moisture management and absorption [10]. Humans - Paper can provide a cleaner air environment in comparison to other particle-based beddings as it does not contain dust. Environment - Paper bedding production can be environmentally friendly and it can be sourced from recycled waste. - Soiled bedding can also be further recycled or composted [10,68].	Horses - Requires more frequent mucking out and is susceptible to mould [10,68]. - Paper is lightweight and may float in windy stables [10,68]. Humans - Paper may be carried by air movement and litter the internal and external environment creating a greater workload. Environment - No disadvantages for the environment were reported in the literature reviewed.
Flax	Horses - Flax is dust- and pest-free, reducing the risk of allergies [9]. - It is highly absorbent and can provide dry bedding. - Flax protects hooves better compared to other bedding materials such as straw [43]. Humans - As only a small amount is required, the cost, handling and storage of the material is reduced [69]. - Flax is conveniently packaged in bags. Environment - Flax is environmentally friendly and compostable [9].	Horses - Flax can increase the risk of colic [69]. - It can be slippery underfoot when first put in place [69]. - The high ammonia concentrations [9] are problematic. Humans - As flax can be initially slippery underfoot, care is required to navigate the fresh bedding surface. Environment - No disadvantages were reported in the literature reviewed.
Hemp	Horses - Hemp is extremely absorbent [9] and can provide dry bedding. - It can have a low dust content [9] and be advantageous for horses with respiratory conditions. Humans - Hemp is easy to muck out and reduces the labour and removal costs [9]. - Hemp is effective in reducing odours. Environment - Hemp is biodegradable and environmentally friendly [9].	Horses - Hemp may cause allergies in sensitive horses. - It can support high ammonia concentrations [9]. Humans - It is an apparent health risk due to its potential high ammonia concentrations. Environment - No disadvantages were reported in the literature reviewed.
Rice hulls	Horses - Rice hulls can be dust-free [9]. - It is a lightweight material [9] and easy to handle.	Horses - They offer poor absorbency and can affect the comfort level including the bedding’s dryness and warmth [9].
Sand	Horses - This material is recommended for horses with laminitis [48]. - Sand bedding helps to reduce house flies development in stables [74]. - It can be easy to clean and affordable [48].	Horses - Ingestion may cause and/or contribute to colic syndrome [75]. Humans - Disposal of sand bedding is challenging as it is not organic [64]. Environment - Although a naturally occurring bedding option, its extraction and disposal are considered environmentally unfriendly [64].
Coconut bedding	Horses - This is highly absorbent and can provide a dry bedding environment [9]. - The material is reported to be dust-free [9]. - It has the lowest ammonia concentration [9]. Environment - Coconut bedding is considered environmentally friendly [9].	Horses - Coconut bedding can encourage the growth of mould and fungi if it becomes too moist and is not properly aerated. - Coconut bedding can be more costly in comparison to more conventional bedding materials like straw, sawdust, or wood shavings
Coffee bedding	Environment - Coffee bedding is considered to be environmentally friendly [77].	Horses - Coffee material is toxic to the horses when ingested [77,78].

## Data Availability

The data presented in this study are available on the AMSActa Institutional Research Repository at the link https://amsacta.unibo.it/cgi/search/simple?q=8264&_action_search=Search&_order=bytitle&basic_srchtype=ALL&_satisfyall=ALL accessed on 5 March 2025.
